# Supplementary material for: Nyctinastic thallus movement in the liverwort Marchantia polymorpha is regulated by a circadian clock
Source: Sci Rep. 2020 May 26;10:8658. doi: 10.1038/s41598-020-65372-8 (PMC7251115; doi:10.1038/s41598-020-65372-8)
Supplement: Supplementary file 1 — Supplementray Information. [file 41598_2020_65372_MOESM1_ESM.pdf]

Nyctinastic thallus movement in the liverwort *Marchantia polymorpha* is regulated by a circadian clock

Ulf Lagercrantz, Anja Billhardt, Sabine N. Rousku, Karin Ljung, D. Magnus Eklund

Supplementary Video S1. Wild-type *Marchantia polymorpha* gemmaling displaying rhythmic circadian movement of thallus lobes. The movie comprise 96 hours at one frame per hour. See separate file.

Supplementary Video S2. Wild-type *Marchantia polymorpha* gemmaling growing on media supplemented with mock (control for videos 3 and 4). See separate file.

Supplementary Video S3. Wild-type *Marchantia polymorpha* gemmaling growing on media supplemented with 10 nM IAA. See separate file.

Supplementary Video S4. Wild-type *Marchantia polymorpha* gemmaling growing on media supplemented with 100 nM IAA. See separate file.

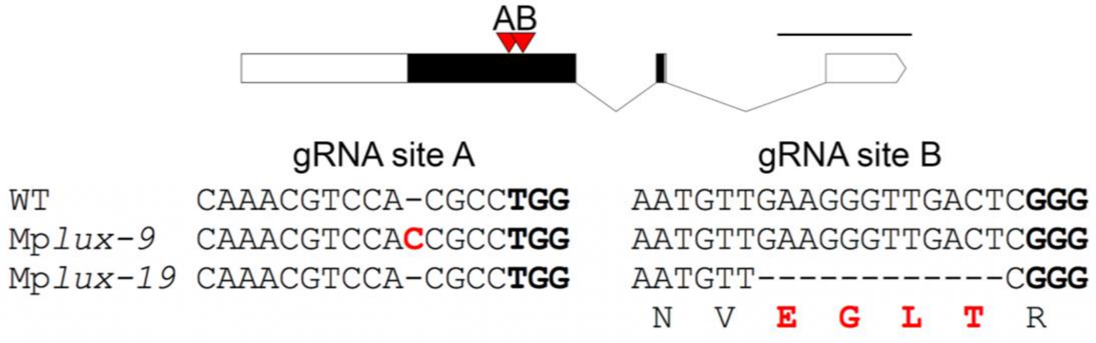

Supplementary Figure S1. Molecular verification of Mp*LUX* loss-of-function mutants. We produced two mutant alleles of the Mp*LUX* locus using two different guide (g) RNAs. Mp*lux*$_{ge}$-9 has an insertion of a cysteine in gRNA site A creating a frame shift mutation upstream of the MYB-domain, and Mp*lux*$_{ge}$-19 has a 12 bp deletion in gRNA site B. The 12 bp (four aa) deletion is located between the DNA-interacting helices two and three of the MYB-domain potentially disrupting their structure and function (Silva et al., 2019), suggesting both alleles could represent complete loss-of-function alleles. Bold letters indicate PAM sites.

Reference

Silva, C.S. et al. (2019). Molecular mechanisms of Evening Complex activity in Arabidopsis. bioRxiv 584854; doi: https://doi.org/10.1101/584854.

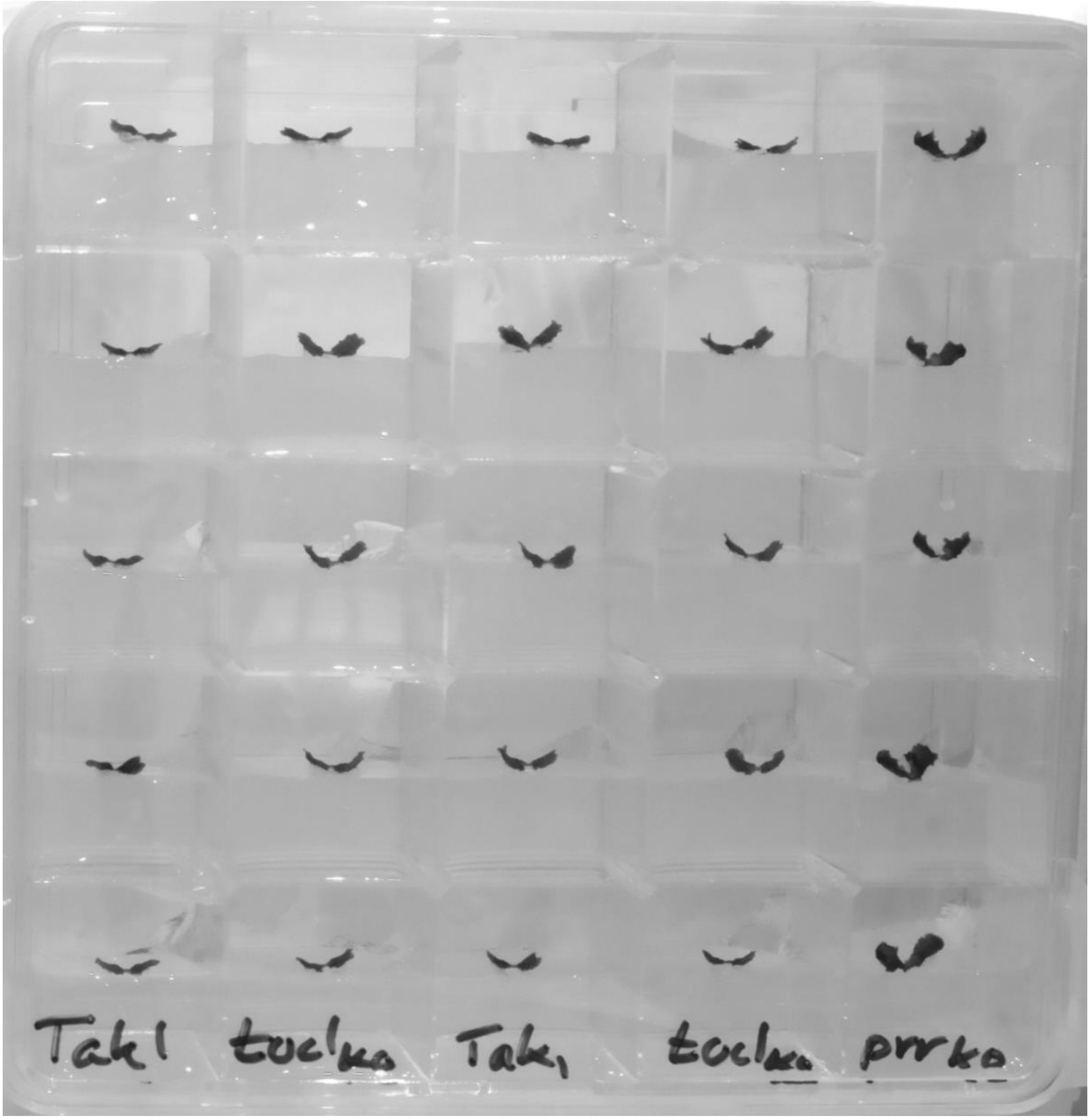

Supplementary Figure S2. *Marchantia polymorpha* gemmalings growing in a vertically positioned 25-well square petri dish. Such plates were placed in a Sanyo growth cabinet for imaging of nyctinastic movements.

Supplementary Table S1. Oligonucleotides used in this study.

Primer name	Sequence - 5' to 3'	Comments
CPEP60	CTCGAACTCTCAAACGTCCACGCC	Frw gRNA A
CPEP61	AAACGGCGTGGACGTTTGAGAGTT	Rev gRNA A
CPCP62	CTCGGAATGTTGAAGGGTTGACTC	Frw gRNA B
CPEP63	AAACGAGTCAACCCTTCAACATTC	Rev gRNA B
ME367	CGAAAGCCCAAGAAGCTACC	Fwd MpAPT qRT-PCR[1]
ME368	GTACCCCCGGTTGCAATAAG	Rev MpAPT qRT-PCR[1]
ME369	AGGCATCTGGTATCCACGAG	Fwd MpACT qRT-PCR[1]
ME370	ACATGGTCGTTCCTCCAGAC	Rev MpACT qRT-PCR[1]
ME402	CTTGGTTGACTTTGGGCAAT	Fwd MpYUC2 qRT-PCR
ME403	CCGACCTTGTCTTTCAGCTC	Rev MpYUC2 qRT-PCR
ME486	GAGATGTTGGGGTTAGTGGAAGTG	Frw MpLUX genotyping
ME487	CCCTTTCTCTCTGATATGCGCTAA	Rev MpLUX genotyping
ME665	CCGAGATCCTGACCAAGG	Frw MpEF1 qRT-PCR[1]
ME666	GAGGTGGGTACTCAGCGAAG	Rev MpEF1 qRT-PCR[1]
ME744	GGACCAAGTGATTCGCTCTC	Frw MpTAA qRT-PCR
ME745	ACAATGCAGCCTGGAAGAGT	Rev MpTAA qRT-PCR
MpPRR F	CAGCAGCTCCTTTGAACAAACA	qRT-PCR[2]
MpPRR R	GCCGTGAAGCAGGAAAGAGAAT	qRT-PCR[2]
MpRVE F	AAACCTCGGCAAAATCAGGAGT	qRT-PCR[2]
MpRVE R	GGCGAGGCAATTTTCAAAGCTG	qRT-PCR[2]
MpTOC1 F	CGAAGGAAGAACGACTGAAGCA	qRT-PCR[2]
MpTOC1 R	TCTGAGACATTTGACGACGACA	qRT-PCR[2]

Notes

1. Saint-Marcoux D, Proust H, Dolan L, Langdale JA. 2015. Identification of reference genes for real-time quantitative PCR experiments in the liverwort *Marchantia polymorpha*. *PLOS ONE* 10: e0118678.
2. Linde A-M, Eklund DM, Kubota A, Pederson ERA, Holm K, Gyllenstrand N, Nishihama R, Cronberg N, Muranaka T, Oyama T, Kohchi T, Lagercrantz U. 2017. Early evolution of the land plant circadian clock. *New Phytologist* 216: 576-590.
